# First Aid Training for Children in Kindergarten: A Pilot Randomized Control Study

**DOI:** 10.3390/children9111626

**Published:** 2022-10-26

**Authors:** Eleana Tse, Katerina Plakitsi, Spyridon Voulgaris, George A. Alexiou

**Affiliations:** 1 Department of Neurosurgery, School of Medicine, University of Ioannina, 451-10 Ioannina, Greece; 2 Department of Early Childhood Education, School of Education, University of Ioannina, 451-10 Ioannina, Greece

**Keywords:** first aid, training, kindergarten children, education

## Abstract

First aid is a fundamental skill for every human of every age, so training in first aid is necessary at a variety of levels. First aid training in schools is essential, but to date, only short reports have been published on the effectiveness of first aid training in kindergarten. We conducted a pilot randomized controlled study on the impact of first aid training on children in kindergarten. We randomly selected 24 children aged 4–5 years from one kindergarten, who were allocated to either a training group (14 children) or a control group (10 children). The training program consisted of three lessons. An eleven-question questionnaire was administered to the children in the training group one day before, one day after, and two and six months after the training, and once to the those in the control group. Before the training, no significant difference was detected in the score on the questionnaire between the two groups. After the lessons, the children in the training group scored significantly higher on the questionnaire than before the lessons, and than the children in the control group. At two and six months after the training, the scores of the children in the training group had decreased but remained higher than before the training and higher that those of the control group. These preliminary results indicate that kindergarten children may benefit from first aid training, but further studies are needed to verify these observations and to explore ways of maintaining the knowledge acquired in training.

## 1. Introduction

First aid is a fundamental skill for every human of every age. Training in first aid is necessary, and analysis has been made of effective and efficient first aid training programs for various populations [1,2,3,4]. Researchers are investigating the effects of first aid training at younger ages and its impact on the capacity of children to provide first aid [5,6,7,8]. Teaching young children at school and in kindergarten can be an effective way to develop first aid capabilities in children of younger ages, and to prevent serious effects from injuries incurred from school accidents. School and kindergarten accidents can be severe, with research indicating that at young ages, the most frequently injured part of the human body is the head [9].

Although many studies have focused on the school training of first aid to children aged from 9 to 18 years, few have been conducted with kindergarten children [10,11]. Bollig and colleagues [12] reported that 4–5-year-old children were able to learn and apply basic first aid and proposed that first aid courses could start at this age, although their study had limitations, such as a small number of participants and no control group. Ammirati and colleagues [13] conducted a comparative study in first aid instruction with trained and untrained kindergarten children, 285 in total. They found that the trained children were more able to describe an emergency and to raise the alarm than those in the control group. A longitudinal cohort study conducted by Banfai and colleagues [14] in kindergarten children demonstrated significant improvement in their knowledge, skills, attitudes, and retention of information up to 15 months post-intervention. They recommended that even though kindergarten children cannot perform cardiopulmonary resuscitation (CPR) correctly, their learning of some aspects of resuscitation is helpful because it is crucial for them to have this knowledge in the case of a real emergency. Plischewski and colleagues [15] evaluated the attitudes of Norwegian kindergarten teachers towards ‘*Henry* first aid training’ and its impact on the understanding of 3–6-year-olds of first aid. They interviewed 92 children who had undergone ‘*Henry* first aid training’ and found that their understanding of first aid had increased post-training. Mohajervatan and colleagues [16], in an observational study, found that kindergarten children can learn urgent first aid, including calling the ambulance service, handling an unconscious patient, and managing severe bleeding.

In view of the few studies which have researched the effectiveness of first aid training in kindergarten children [17], this pilot study aimed to examine the effect of teaching first aid to children aged from 4 to 5 years in a Greek kindergarten. The research questions that this study attempted to answer were:

1. Are the kindergarten children able to learn first aid?
2. How long can the kindergarten children retain first aid knowledge?

## 2. Materials and Methods

We performed a randomized controlled trial. First, one kindergarten was randomly selected, and then the 4–5-year-old children were divided into two groups, the training group, who followed a course in first aid, and the control group. We requested the participation of 24 children and asked for written, informed consent from their parents. The control group consisted of 10 children and the training group of 14 children. All the children in the training group attended a course of three first-aid lessons and participated in follow-up evaluation. We administered a questionnaire to the children in the training group one day before, one day after, and two and six months after the training program. The questionnaire was administered to the children in the control group once, at the start of the study. The questionnaire consisted of eleven questions: two questions related to demographic characteristics and nine first aid questions, specifically, eight multiple-choice questions and one time-ordered questions (Appendix A). The researchers did not access individual children’s data, according to the National Education System’s confidentiality regulation. The study was registered as a Clinical Trial (NCT05563129).

### 2.1. Ethical Considerations

The Ethics Department of the University of Ioannina approved the research program before it was conducted. The participants, their parents, and the teachers involved received written and oral information before the start of the study. The researcher informed the parents of their right to withdraw their child at any time, with no personal consequences, and assured them of anonymity. The parents provided written informed consent for their children to enter the study.

### 2.2. Training Program

The training program consisted of three lessons (45 min/lesson) given on three consecutive days, and it contained theoretical (electronic presentations) and role-play training. We selected specialized first aid staff with kindergarten training experience to conduct the lessons. The subjects taught were: what is first aid, the rules of first aid, calling the ambulance (the telephone number to call, when you should contact an ambulance, how to provide the correct information), and how to deal with a head injury, nose bleeding, bleeding from a cut, and choking. We adapted the training program according to the age group and the frequency of everyday accidents in that group.

### 2.3. Statistical Analysis

The data are presented as percentages (%) with 95% confidence interval (CI). Statistical analysis of the results was performed using the *t*-test (significance level: *p* < 0.05). Descriptive statistics were calculated (percentages, means).

## 3. Results

The study included 24 children, 13 girls and 11 boys, with a median age of 5 years. The training group consisted of 14 children, and the control group of 10 children. We evaluated the training group using the questionnaire, one day pre-training, one-day post-training, and two and six months after training. In addition, we assessed the control group once. The comparative results are shown in Table 1.

Before the training, the children in the training and control groups showed no significant difference in their level of knowledge of first aid procedures. One day after the training, the children in the training group scored significantly higher than the children in the control group (Table 1). After two and six months post-training, this knowledge had declined, but remained higher than before the training, and higher than that of the control group. After the first aid course, most children were able to give the correct emergency telephone number and to know when they should call the ambulance and what information they should give to the rescuers. The majority of children learned how they can manage bleeding and minor head injury. Specifically, according to the results shown in Table 2, one day post-training, 100% of children in the training group knew how to deal with a minor head injury and bleeding correctly; 93% answered correctly the reasons for calling the ambulance; 86% had learned how to deal with nose bleeding and 86% knew the correct emergency telephone number; 50% knew who should hang up the phone first when the ambulance is reached; 71% gave correct answers to questions about trauma and the first reaction of choking; and 64% knew how to call an ambulance. In Table 2, the results of day 1 pre-post-training and 2- and 6-months post-training are presented. After 6 months, the knowledge of most children about how to manage nose bleeding and trauma had declined significantly.

## 4. Discussion

The findings of this study showed that five-year-old children can learn basic first aid, including calling the ambulance, and managing trauma, head injury, bleeding, nose bleeding, and choking. This finding is in consonance with the earlier studies described in the introduction [12,15,16]. Even at 2 and 6 months after the 3-day training program, most of the acquired knowledge of the children in the training was retained and was significantly better than their pre-training level of first aid knowledge and that of the control group. This finding agrees with that of Banfai and colleagues [14]. The follow-up interviews showed that some of the children in the training group had forgotten some of the information, thus increasing the percentage of wrong answers compared with that immediately after the initial training. The 2 and 6 months test results, which remained higher than those in the initial test and higher than the results of the control group, is in agreement with the studies of Banfai and colleagues [14] and of Ammirati and colleagues [13]. It is apparent that teaching first aid to kindergarten children using scenario-based methods with both practical and theoretical components helped them to learn first aid. This finding is in accordance with the review of Reveruzzi, Buckley, and Sheehan [1].

Regarding the training of the children in dealing with head injuries, no study has explored the impact of this type of training, even though head injuries are the most common in pre-school and early school [9]. Our study focused on the recognition and management of head injuries, because this is the most common type of injury in the kindergarten and primary school setting. Although many researchers have focused on the frequency and impact of generic first training in schools, there is no report of first aid training centered on head injuries. This study therefore attempted to enrich the scientific research on this topic.

The training we conducted, and especially the scenario-based sessions, created an atmosphere of enthusiasm among the children and their teachers and parents. The teachers and parents commented on the need for first aid training for everyone, including children. It is of note that one kindergarten teacher commented that the sessions refreshed her knowledge of the correct first aid procedures, confirming the fact that teachers also need recurrent first aid training, as several studies have shown [15].

In relation to our study, we conducted a systematic review of the effectiveness of resuscitative or non-resuscitative first-aid training for primary school children, based on 10 articles. This review showed that children aged under 11 years can learn the theoretical knowledge of CPR but cannot perform CPR correctly because they are not strong enough to achieve the proper depth of chest compressions, and because of their anthropometric characteristics (weight, height, and body mass index). Practical training in CPR should be provided at a later age.

School, including kindergarten, is an appropriate place to educate people on first aid, because it assembles many people and they can be trained cost-effectively. It is essential to teach first aid at schools every year in order to maintain the level of acquired knowledge and skills. Increasing the number of first aid trained bystanders will reduce the number of accidental deaths. Teaching first aid to children from an early age can cultivate social responsibility, which is necessary for the progress of society [10,17]. To conclude, we firmly believe that educating young children in both a resuscitative and non-resuscitative first aid program is crucial.

## 5. Conclusions

Many researchers have focused on teaching first aid to schoolchildren at older ages [3,6,18]. This pilot study examined the effects of teaching first aid to 4–5-year-old children in kindergarten, with promising results. The limitations of our study were the small sample size and that the control group was not tested over time and there may have been change if they were all in the same school. Finally, the survey was not validated and may not be useful in other kindergarten populations. Nevertheless, analysis of the study results showed that scenario-based first aid training could develop the first aid knowledge of kindergarten children, and that the children retain some of this knowledge even 6 months after the training. It needs to be explored how often the first aid training of kindergarten and schoolchildren should be repeated, along with the optimal methods of training children on trauma management. There is a need for further research in these areas.

## Figures and Tables

**Table 1:** Comparison of the level of knowledge of first aid in a training group of kindergarten children (n = 14) and a control group (n = 10).

Comparison between Groups	*p* Value
Control vs. pre-training	0.3634
Control vs. post training at day 1	<0.0001
Control vs. follow-up 2 months	<0.0001
Control vs. follow-up 6 months	0.0008
Pre-training vs. post-training at day 1	0.0002
Pre-training vs. follow-up 2 months	0.0002
Pre-training vs. follow-up 6 months	0.0066
Post-training at day 1 vs. follow-up 2 months	0.7
Post-training at day 1 vs. follow-up 6 months	0.12

**Table 2:** Rate (%) of correct answers to the first aid questionnaire of kindergarten children: training group (n = 14) and control group (n = 10).

Topic	Activity	Pre-Training	Post-Training	After 2 Months	After 6 Months	Control Group
Call the ambulance	Correct telephone number	64	86	64	79	40
	Correct reason to call the ambulance	93	93	93	100	70
	Correct information given to the rescuers	26	64	78	76	30
	Correct actions to call the ambulance	36	50	36	36	30
Manage bleeding	Correct reaction to nose bleeding	21	86	71	26	10
	Correct reaction to minor bleeding	64	100	100	93	50
Choking	Correct reaction to choking	21	71	71	57	30
Injury	Correct reaction to head injury	76	100	93	86	70
	Correct reaction to trauma	0	71	64	43	10

## Appendix A

Questionnaire

Please provide your gender and age and answer the questions below. If you do not know an answer, please select the answer you think is correct.

1. Gender:
2. Age: 4 5 6
3. A severe accident happens in front of you, and you must call an ambulance. What do you have to do? The pictures below show the actions you need to take, but they are in random order. Put them in the correct order by filling in the numbers 1 to 4.
4. What would you do if your friend hurts her/his head lightly?

Circle the correct answer.
  a. You immediately tell your friend to get up and continue your game.
  b. You put toothpaste on your friend’s wound.
  c. You help your friend sit on a chair and ask if he/she is dizzy.

5. What would you do if, while playing with your friend, he/she gets injured severely?

Circle the correct answer.
  a. You help your friend get up.
  b. You run away to avoid punishment.
  c. You call for help and do not move him/her.

6. What would you do if your friend injures his/her palm and the wound is slightly bleeding?

Circle the correct answer.
  a. You apply pressure to the wound with a gauze pad or clean cloth.
  b. You wipe the blood with your hand.
  c. You apply toothpaste to the wound.

7. What would you do if you got a nosebleed?

Circle the correct answer.
  a. You would call an ambulance.
  b. You would sit on a chair with your head facing down.
  c. You would hold your head up to stop bleeding.

8. When calling an ambulance, you must press:

Circle the correct answer.
  a. 100
  b. 166
  c. 199

9. Would you call the ambulance if:

Circle the correct answer.
  a. Someone is injured lightly on the hand.
  b. Someone was severely injured on the head.
  c. Someone is injured lightly on the leg.

10. What should you say on the phone when you call an ambulance?

Circle the correct answer.
  a. Your name and description of the accident.
  b. Describe the accident.
  c. Your name, the location of the accident, a description of the accident, and the number of injured people

11. What would you do if your friend chokes while eating his/her food?

Circle the correct answer.
  a. You give your friend water.
  b. You leave your friend alone.
  c. You slap your friend on the back.

Thank you for completing this questionnaire!
